# Unveiling the tick-borne pathogens from domestic ruminant ticks in Malawi and the emergence of the brown ear tick in the southern region: implications for East Coast fever control

**DOI:** 10.1051/parasite/2026022

**Published:** 2026-04-17

**Authors:** Boniface Chikufenji, Uday Kumar Mohanta, Elisha Chatanga, Dallion Stopher, Onur Ceylan, Rika Umemiya-Shirafuji, Oriel Thekisoe, Madalitso Nkhata, Tatsuki Sugi, Junya Yamagishi, Xuenan Xuan, Kyoko Hayashida

**Affiliations:** 1 Division of Collaboration and Education, International Institute for Zoonosis Control, Hokkaido University Kita-20, Nishi-10 Sapporo Hokkaido 001-0020 Japan; 2 Ministry of Agriculture, Department of Animal Health and Livestock Development P. O. Box 2096 Lilongwe Malawi; 3 Department of Microbiology and Parasitology, Sher–e–Bangla Agricultural University Sher–e–Bangla Nagar Dhaka-1207 Bangladesh; 4 Department of Veterinary Pathobiology, Faculty of Veterinary Medicine, Lilongwe University of Agriculture and Natural Resources P. O. Box 219 Lilongwe Malawi; 5 Department of Veterinary Parasitology, Faculty of Veterinary Medicine, Selcuk University Konya-42130 Türkiye; 6 National Research Center for Protozoan Diseases, Obihiro University of Agriculture and Veterinary Medicine Obihiro Hokkaido 080-8555 Japan; 7 Unit of Environmental Sciences and Management, North-West University Potchefstroom 2531 South Africa; 8 Vets of Purpose Organization P.O. Box 2355 Lilongwe Malawi; 9 Research Centre for Asian Infectious Diseases, Institute of Medical Science, The University of Tokyo 4-6-1 Shirokanedai, Minato-ku Tokyo 108-8639 Japan

**Keywords:** East Coast fever, Malawi, Molecular detection, *Rhipicephalus appendiculatus*, *Theileria parva*, tick species diversity

## Abstract

Ticks transmit a wide range of protozoan, bacterial, and viral pathogens to humans and animals globally. However, data on ticks infesting domestic ruminants and the pathogens they carry are scarce in Malawi. In this study, we examined ticks collected from domestic ruminants and screened them for selected veterinary and medically important protozoan and bacterial pathogens. A total of 964 ticks were collected from 202 cattle, 63 goats, and 16 sheep across eleven districts in Malawi. Ticks were morphologically identified to species level using taxonomical keys, with molecular confirmation by PCR amplification and sequencing of the 12S ribosomal RNA (12S rDNA) and cytochrome c oxidase subunit I (*COI*) genes. Tick DNA was further screened for tick-borne pathogens using species-specific PCR assays. Identified tick species included *Rhipicephalus microplus* (30.5%), *Rhipicephalus appendiculatus* (23.3%), *Rhipicephalus decoloratus* (13.2%), *Rhipicephalus evertsi* (9.8%), *Hyalomma rufipes* (7.5%), *Amblyomma variegatum* (6.3%), *Rhipicephalus sanguineus* sensu lato (tropical lineage) (3.6%), *Hyalomma truncatum* (2.8%), *Rhipicephalus simus* (2.0%), *Rhipicephalus pravus* (0.6%), and *Rhipicephalus annulatus* (0.4%). Overall, 37.0% of ticks carried at least one tick-borne pathogen, with *Theileria parva* being the most prevalent (34.7%), followed by *Anaplasma marginale* (17.4%), *Babesia bigemina* (14.9%), *Anaplasma ovis* (11.2%), *Ehrlichia ruminantium* (9.2%)*, Theileria mutans* (8.4%), *Babesia bovis* (2.2%), and *Anaplasma bovis* (2.0%). This study provides the first molecular identification of ticks infesting domestic ruminants in Malawi and documents associated tick-borne pathogens. Notably, *Rhipicephalus appendiculatus* was identified for the first time in southern Malawi, refining current understanding of East Coast fever epidemiology and highlighting the need for updated surveillance approaches.

## Abstract (fr)

Les tiques transmettent un large éventail d'agents pathogènes protozoaires, bactériens et viraux aux humains et aux animaux à l'échelle mondiale. Cependant, les données sur les tiques infestant les ruminants domestiques et les agents pathogènes qu'elles transportent sont rares au Malawi. Cette étude a examiné des tiques prélevées sur des ruminants domestiques et a recherché certains agents pathogènes protozoaires et bactériens d'importance vétérinaire et médicale. Au total, 964 tiques ont été collectées sur 202 bovins, 63 chèvres et 16 moutons dans onze districts du Malawi. Les tiques ont été identifiées morphologiquement jusqu'à l'espèce à l'aide de clés taxonomiques, avec confirmation moléculaire par amplification PCR et séquençage des gènes de l'ARN ribosomique 12S (ADNr 12S) et de la sous-unité I de la cytochrome c oxydase (*COI*). L’ADN des tiques a ensuite été analysé à la recherche d’agents pathogènes transmis par les tiques à l’aide de tests PCR spécifiques à l’espèce. Les espèces de tiques identifiées comprenaient *Rhipicephalus microplus* (30,5 %), *Rhipicephalus appendiculatus* (23,3 %), *Rhipicephalus decoloratus* (13,2 %), *Rhipicephalus evertsi* (9,8 %), *Hyalomma rufipes* (7,5 %), *Amblyomma variegatum* (6,3 %), *Rhipicephalus sanguineus sensu lato* (lignée tropicale) (3,6 %), *Hyalomma truncatum* (2,8 %), *Rhipicephalus simus* (2,0 %), *Rhipicephalus pravus* (0,6 %) et *Rhipicephalus annulatus* (0,4 %). Au total, 37 % des tiques étaient porteuses d'au moins un agent pathogène transmis par les tiques, *Theileria parva* étant le plus fréquent (34,7 %), suivi d'*Anaplasma marginale* (17,4 %), *Babesia bigemina* (14,9 %), *Anaplasma ovis* (11,2 %), *Ehrlichia ruminantium* (9,2 %), *Theileria mutans* (8,4 %), *Babesia bovis* (2,2 %) et *Anaplasma bovis* (2 %). Cette étude fournit la première identification moléculaire des tiques infestant les ruminants domestiques au Malawi et documente les agents pathogènes transmis par les tiques associés. Notamment, *Rhipicephalus appendiculatus* a été identifié pour la première fois dans le sud du Malawi, ce qui affine les connaissances actuelles sur l'épidémiologie de la fièvre de la côte Est et souligne la nécessité de mettre à jour les méthodes de surveillance.

## Introduction

Ticks affect a wide range of hosts, including humans, livestock, and wildlife and transmit pathogens causing protozoan, viral, and bacterial diseases such as theileriosis, Crimean-Congo hemorrhagic fever, hepatozoonosis, babesiosis, anaplasmosis, and heartwater [12, 34, 45]. The wounds inflicted on the animal body by some ticks, like *Amblyomma variegatum* during feeding, may become a portal of entry for the pathogens causing secondary bacterial infections such as dermatophilosis [43]. Further, ticks suck a large volume of blood from their hosts [22], resulting in discomfort [41], skin irritation [39], anemia, and excretion of toxins in their saliva [40].

Most of the global cattle population is at significant risk of tick infestation with approximately 80% of the world’s cattle exposed to this threat. Ticks are widespread and thrive in various climates, particularly in tropical and subtropical regions where most cattle are reared [22]. Ticks and tick-borne diseases (TBDs) cause substantial economic losses in the livestock industry, with annual losses estimated at around US$ 22–30 billion globally [23, 32]. The tick challenge causes great economic losses, especially in many resource-limited countries of Africa, where much of the resources are routed towards poverty and hunger reduction rather than fighting ticks and TBDs. Ticks also have a large impact on public health due to the pathogens they carry and potentially transmit to humans, causing zoonotic TBDs [12].

In Malawi, 80% of the total population are farmers, of which about 60% own livestock such as cattle, goats, sheep, pigs, poultry, rabbits, and other non-conventional livestock species [33]. The domestic ruminant population is estimated at 2 million cattle, 12 million goats, and 400,000 sheep [33]. The contribution of the livestock subsector to Malawi’s economy and nutrition is substantial, accounting for 10.5% to the national gross domestic product (GDP) and 37.4% to the agricultural GDP [33].

Tick-borne pathogens (TBPs) belonging to genera *Anaplasma*, *Babesia*, *Theileria*, *Ehrlichia*, and *Rickettsia* have been implicated in causing TBDs and related deaths in livestock in Malawi [6]. The previous study focused on the host animal [6], and pathogens carried by ticks infesting dogs [7], while data on pathogens carried by ticks infesting domestic ruminants remain limited. Further, previous work in Malawi detected *Theileria parva* in cattle blood [8], yet the principal vector of *T. parva*, *Rhipicephalus appendiculatus* was not believed to exist in southern Malawi [9]. This paradox raised epidemiological questions which our current study resolves by confirming the presence of *R. appendiculatus* in this region for the first time.

## Materials and methods

### Ethics

Tick collection was performed with ethical approval from Animal Health Committee of the Department of Animal Health and Livestock Development (DAHLD) in the Ministry of Agriculture in Malawi (AHC/DAHLD 002/2022) and prior consent was sought from the animal owners.

### Study area and tick collection

**Figure 1:**
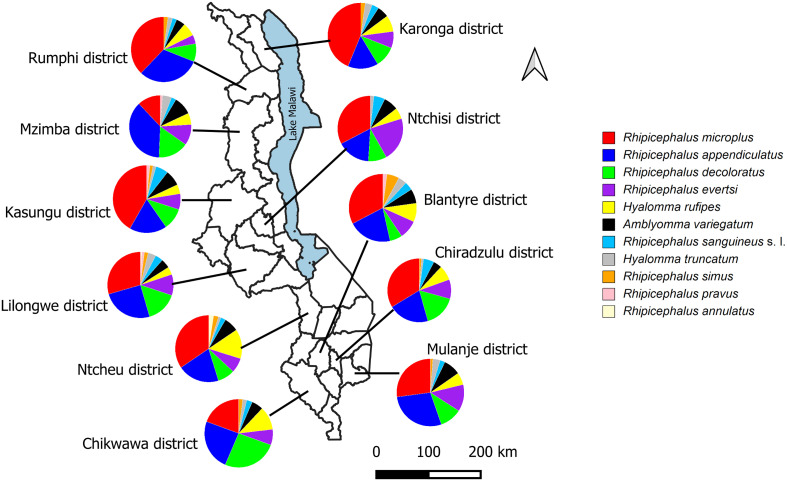
Map of Malawi showing sampling sites and tick species identified at each location. The map was generated using QGIS software version 3.40.1.

Ticks were collected between February 2022 and December 2023 from apparently healthy but infested cattle (*n* = 202), goats (*n* = 63), and sheep (*n* = 16) in 11 districts of Malawi, namely, Chikwawa (16.0438° S, 34.8017° E), Chiradzulu (15.6759° S, 35.1406° E), Mulanje (16.0252° S, 35.5083° E), and Blantyre (15.7667° S, 35.0168° E) in the southern region, Ntcheu (14.8220° S, 34.6359° E), Lilongwe (13.9626° S, 33.7741° E), Kasungu (13.0357° S, 33.4720° E), and Ntchisi (13.2842° S, 33.8858° E) in the central region, and Mzimba (11.8992° S, 33.5924° E), Karonga (9.9525° S, 33.9248° E), and Rumphi (11.0172° S, 33.8551° E) in the northern region (Figure 1). A total of 964 ticks were collected from cattle (*n* = 784), goats (*n* = 151), and sheep (*n* = 29) by brushing the fur and using forceps to remove the ticks. Ticks were collected from all the animal parts, including the ear pinnae, neck region, and under the tail. The collected ticks were individually stored in 1.5 mL microcentrifuge tubes containing 70% ethanol for preservation until further processing.

### Morphological identification of ticks

Ticks were identified using a stereomicroscope (Olympus SZX16, Tokyo, Japan) following published taxonomical keys [29, 48]. The ticks were segregated based on sex as male or female, and based on developmental stage as adults, nymphs, and larvae. Only intact adult ticks were identified to species level morphologically, while the immature and damaged ticks were identified to species level using molecular methods.

### DNA extraction

The individual ticks preserved in 70% ethanol were washed with distilled water and were later air-dried. Out of the 964 ticks collected, 963 ticks were processed individually for genomic DNA extraction. Only one pooled sample was prepared, consisting of 16 adult female ticks and 17 larvae of the same tick species (*R. appendiculatus*), all collected from a single host animal (cattle), and treated as one sample for DNA extraction. Liquid nitrogen was used to freeze the ticks in the tubes for easy crushing with a sterile pestle. Thereafter, DNA was extracted using a NucleoSpin Tissue DNA extraction kit (Macherey-Nagel, Düren, Germany), following the manufacturer’s guidelines. The DNA was eluted with 50 μL of elution buffer, and the concentration was measured using a NanoDrop spectrophotometer (Thermo Fisher Scientific, Waltham, MA, USA). The extracted DNA was stored at −30 °C until required for use.

### Genetic identification of ticks and detection of tick-borne pathogens

A total of 964 ticks were collected and processed for molecular analyses. DNA was extracted from individual ticks, except for 16 adult tick samples and 17 larvae that were processed as pooled sample, comprising ticks of the same species, developmental stage, and host origin. For tick species confirmation, representative DNA samples from individual ticks were subjected to PCR amplification and sequencing of the mitochondrial *12S rRNA* and/or *COI* markers using the primers listed in Table 1. All DNA extracts, including those from the pooled sample, were subsequently screened for selected TBPs using species-specific PCR assays (Table 1). A total reaction volume of 10 μL containing 0.2 μL of each 10 μM primer, 2 μL of a 5× Standard buffer (New England Biolabs, Ipswich, MA, USA), 0.2 μL of 10 mM deoxynucleotide triphosphate mix (dNTPs), 0.05 μL of *Taq* polymerase (New England Biolabs), 2 μL of template DNA, and 5.35 μL of UltraPure water (Invitrogen, Waltham, MA, USA), was used for PCR assays. The PCR reactions were performed in a thermal cycler (VeritiPro, Applied Biosystems, Foster City, CA, USA), with conditions obtained from previous studies (Table 1). UltraPure water was used as a negative control for quality control, and previously confirmed PCR-positive samples were used as the positive controls. Electrophoresis of PCR products was carried out using a 1.5% agarose gel, stained with ethidium bromide solution and later viewed under an Ultraviolet (UV) transilluminator (Atto Corporation, Tokyo, Japan). The PCR amplicons from the gel were extracted and purified using a NucleoSpin Gel and PCR Clean-up kit (Macherey-Nagel, Düren, Germany), following the manufacturer’s guidelines. Direct sequencing was performed using BigDye Terminator Cycle sequencing kit (Applied Biosystems) and ABI Prism 3100 Genetic Analyzer (Applied Biosystems).

**Table 1:** Primer list used for tick identification and detection of tick-borne pathogens in ruminant-infesting ticks from Malawi.

Pathogen (gene)	Assays	5'→3'	Product base size (bp)	References
*T. parva* (*p104*)	PCR	ATTTAAGGAACCTGACGTGACTGC	496	[37]
		TAAGATGCCGACTATTAAATGAC		
	nPCR	GGCCAAGGTCTCCTTCAGAATACG	277	
		TGGGTGTGTTTCCTCGTCATCTGC		
*B. bovis* (*sbp-4*)	PCR	AGTTGTTGGAGGAGGCTAAT	907	[47]
		TCCTTCTCGGCGTCCTTTTC		
	nPCR	GAAATCCCTGTTCCAGAG	503	
		TCGTTGATAACACTGCAA		
*B. bigemina* (*rap-1a*)	PCR	GAGTCTGCCAAATCCTTAC	879	[47]
		TCCTCTACAGCTGCTTCG		
	nPCR	AGCTTGCTTTCACAACTCGCC	412	
		TTGGTGCTTTGACCGACGACA		
*T. mutans* (18S rRNA)	PCR	GACACAGGGAGGTAGTGACAAG	403	[42]
		CTAAGAATTTCACCTCTGACAGT		
	snPCR	AACATTCGGAGACGCAAGCGAG	258	
*A. marginale* (*groEL*)	PCR	GACTACCACATGCTCCATACTG	866	[49]
		GTCTGAAGATGAGATTGCACAGGT		
	nPCR	GACGTCCACAACTACTGCATTCA	~768	
		CCTTTGATGCCGTCCAGAGATGCA		
Tick (CO1)	PCR	CTTCAGCCATTTTACCGCGA	793	[28]
		CTCCGCCTGAAGGGTCAAA		
*E. ruminantium* (16S rRNA)	PCR	GGTACCTACAGAAGAAGTCC	1489	[35]
		TAGCACTCATCGTTTACAGC		
Tick (12S rRNA)	PCR	AAACTAGGATTAGATACCCTATTA	406	[31]
		CTATGTAACGACTTATCTTAATAA		
*A. bovis* (16S rRNA)	PCR	CTCGTAGCTTGCTATGAGAAC	551	[21]
		TCTCCCGGACTCCAGTCTG		
*A. ovis* (*msp4*)	PCR	CCGGATCCTTAGCTGAACAGGAAT	347	[49]
		GGGAGCTCCTATGAATTACAGAGA		

PCR = Polymerase Chain Reaction; nPCR = Nested PCR; snPCR = Semi-nested PCR

### Phylogenetic analysis and accession numbers

The sequences obtained were assembled, edited, and trimmed using Codon Code Aligner version 9 software (Codon Code Corporation, Centerville, MA, USA). The consensus sequences were compared with reference sequences available in GenBank using the nucleotide Basic Local Alignment Search Tool (BLASTn) of the National Center for Biotechnology Information (NCBI) to confirm preliminary identity. Multiple sequence alignments were performed using the ClustalW algorithm implemented in Mega XI software [46]. The best-fitting nucleotide substitution model for each gene dataset was determined based on the Bayesian information Criterion (BIC) in MEGA XI. Phylogenetic trees were constructed using the Maximum Likelihood (ML) method with 1,000 bootstrap replicates to assess node support. Bootstrap values ≥50% were considered significant and are shown on the corresponding nodes. Appropriate outgroup sequences were selected for each analysis based on published literature and GenBank annotations. The new sequences were deposited in GenBank with accession numbers OR616666–OR616670 (*R. microplus*), OR616672–OR616675 (*R. decoloratus*), OR616700–OR616704 (*R. appendiculatus*), OR635619–OR635625 (*R. evertsi*), OR635443–OR635447 (*H. truncatum*), OR635234–OR635238 (*H. rufipes*), OR727797–OR727801 (*R. sanguineus* s. l*.*), OR842983–OR842987 (*A. variegatum*), OR843043–OR843046 (*R. annulatus*), OR843047– OR843048 (*R. pravus*), OR843050–OR843052 (*R. simus*), OR755894– OR755898 (*T. mutans*), OR767908– OR767912 (*B. bigemina*), OR767904– OR767907 (*A. marginale*), OR767913– OR767917 (*T. parva*), OR819704– OR818708 (*A. ovis*), OR818699– OR818703 (*B. bovis*), OR821752– OR821755 (*E. ruminantium*), and OR823813– OR823817 (*A. bovis*).

## Results

### Tick species identification

We collected ticks exclusively from animals that were found to be visibly infested at the time of sampling. From each infested animal, 1–10 ticks were collected depending on the infestation intensity. Accordingly, we sampled 784 ticks (324 adult males, 391 adult females, 52 nymphs, and 17 larvae) from 202 heads of cattle, 151 ticks (82 adult males, 54 adult females, 11 nymphs, and 4 larvae) from 63 goats, and 29 ticks (9 adult males, 18 adult females, and 2 nymphs) from 16 sheep, totaling to 964 ticks (Table 2; Table S1). Of the sampled ticks, 41.2% were from the central region, 32.1% from the southern region, and 26.7% from the northern region. Ticks were morphologically identified and classified into three genera: *Amblyomma*, *Hyalomma*, and *Rhipicephalus*. Eleven tick species were further identified including *R. microplus* (30.5%), *R. appendiculatus* (23.3%), *R. decoloratus* (13.2%), *R. evertsi* (9.8%), *H. rufipes* (7.5%), *A. variegatum* (6.3%), *R. sanguineus* s. l. (3.6%), *H. truncatum* (2.8%), *R. simus* (2.0%), *R. pravus* (0.6%), and *R. annulatus* (0.4%) (Fig. 1). All the tick species were found to be distributed in all the districts except *R. annulatus,* which was only found in Ntcheu and Lilongwe districts (Fig. 1). Notably, *R. appendiculatus* was detected in southern districts (Chikwawa, Blantyre, Chiradzulu, and Mulanje), contradicting previous reports that the region was free of this vector. The PCR sequence analysis based on *12S rRNA* and/or *COI* genes showed perfect agreement between molecular and morphological identification. The tick sequences obtained in this study showed identities ranging between 96% and 100% when compared to those in the GenBank database, supporting the accuracy of the morphological identification.

**Table 2:** Tick species and associated tick-borne pathogens identified in different host animals in Malawi.

Tick species	Tick number	Animal spp.	Number and prevalence of ticks positive for each tick-borne pathogen								
			*A. mar* (%)	*A. ovis* (%)	*A. bovis* (%)	*B. big* (%)	*B. bovis* (%)	*T. mutans* (%)	*T. parva* (%)	*E. rum* (%)	Total (%)

*Amblyomma* sp. (*n* = 61)											
*A. variegatum*	61	Cattle Goats Sheep	0	0	0	0	0	0	0	24 (39.3)	24 (39.3)
*Hyalomma* sp. (*n* = 99)											
*H. truncatum*	27	Cattle Goats Sheep	3 (11.1)	14 (51.8)	0	0	0	0	0	9 (33.3)	26 (96.3)
*H. rufipes*	72	Cattle Goats Sheep	8 (11.1)	0	0	0	0	0	0	0	8 (11.1)
*Rhipicephalus* sp. (*n* = 804)											
*R. decoloratus*	127	Cattle Goats Sheep	0	0	0	31 (24.4)	0	12 (9.5)	37 (29.1)	0	80 (63.0)
*R. evertsi*	94	Cattle	11 (11.7)	7 (7.5)	0	0	0	9 (9.6)	16 (17.0)	0	43 (45.7)
*R. appendiculatus*	225	Cattle Goats Sheep	26 (11.6)	19 (8.4)	0	22 (9.8)	5 (2.2)	0	69 (30.7)	0	141 (62.7)
*R. microplus*	294	Cattle Goats Sheep	9 (3.1)	0	0	0	3 (1.0)	0	0	0	12 (4.1)
*R. annulatus*	4	Goats	0	0	1 (25.0)	0	0	0	0	0	1 (25.0)
*R. sanguineus* s. l.	35	Cattle	5 (14.3)	0	0	0	0	0	0	0	5 (14.3)
*R. simus*	19	Cattle	0	0	6 (31.6)	0	0	9 (47.4)	0	0	15 (79.0)
*R. pravus*	6	Cattle	0	0	0	0	0	0	2 (33.3)	0	2 (33.3)
**Total**	**964**		**62 (6.4)**	**40 (4.2)**	**7 (0.7)**	**53 (5.5)**	**8 (0.8)**	**30 (3.1)**	**124 (12.9)**	**33 (3.4)**	**357 (37.0)**

*A. mar = Anaplasma marginale*; *B. big = Babesia bigemina*; *E. rum = Ehrlichia ruminantium*

Values indicate the absolute number of PCR-positive ticks.

### Tick-borne pathogen detection

Selected TBPs were screened from the identified ticks, and a total of eight pathogens were detected in the ticks, with no TBP detected in the pooled tick sample. A total of 357 (37.0%) ticks were positive for at least one of the TBPs. Among the tick DNA samples that tested positive, *T. parva* was the most frequently detected pathogen (124/357, 34.7%), followed by *A. marginale* (62/357, 17.4%), *B. bigemina* (53/357, 14.9%)*, A. ovis* (40/357, 11.2%)*, E. ruminantium* (33/357, 9.2%), and *T. mutans* (30/357*,* 8.4%). In contrast, *B. bovis* (8/357, 2.2%) and *A. bovis* (7/357, 2.0%) were detected at relatively low frequencies (Table 3). *Theileria parva* was detected in *R. appendiculatus*, *R. decoloratus*, *R. evertsi*, and *R. pravus* ticks, whereas *A. marginale* was detected in *H. truncatum*, *H. rufipes*, *R. evertsi*, *R. appendiculatus*, *R. microplus*, and *R. sanguineus* s. l. Moreover, *B. bigemina* was detected in *R. decoloratus* and *R. appendiculatus*. *Anaplasma ovis* was detected in *H. truncatum*, *R. evertsi*, and *R. appendiculatus*. *Ehrlichia ruminantium* was detected in *A. variegatum* and *H. truncatum*, while *Theileria mutans* was detected in *R. decoloratus*, *R. evertsi*, and *R. simus*, and *Anaplasma bovis* was detected in *R. annulatus* and *R. simus.* Furthermore, *B. bovis* was detected in *R. appendiculatus* and *R. microplus* (Table 2). Regarding sex and development stages, 47.3% of the male ticks, 50.1% of the female ticks, and 2.6% of the nymphs were positive for at least one of the TBPs screened.

**Table 3:** Tick-borne pathogen detection rates in ticks by district in Malawi.

Pathogen	District											
	Chikwawa (%)	Chiradzulu (%)	Mulanje (%)	Blantyre (%)	Ntcheu (%)	Lilongwe (%)	Kasungu (%)	Ntchisi (%)	Mzimba (%)	Rumphi (%)	Karonga (%)	Total ticks (%)
*A. marginale*	6 (22.2)	11 (39.3)	3 (15.8)	9 (18.0)	2 (6.7)	4 (5.4)	5 (12.5)	2 (10.5)	7 (13.2)	4 (13.8)	9 (23.1)	62 (17.4)
*A. bovis*	0	0	0	0	7 (19.4)	0	0	0	0	0	0	7 (2.0)
*A. ovis*	8 (29.6)	4 (14.3)	2 (10.5)	4 (8.0)	3 (10.0)	3 (4.1)	4 (10.0)	0	5 (9.4)	3 (10.3)	4 (10.3)	40 (11.2)
*B. bigemina*	0	2 (7.1)	1 (5.3)	5 (10.0)	3 (10.0)	12 (16.2)	7 (17.5)	4 (21.1)	7 (13.2)	8 (27.6)	4 (10.3)	53 (14.9)
*B. bovis*	0	0	5 (21.7)	0	0	2 (2.7)	0	0	0	1 (3.4)	0	8 (2.2)
*T. parva*	3 (11.1)	4 (14.3)	0	7 (14.0)	11 (36.7)	27 (36.5)	19 (47.5)	12 (63.2)	21 (39.6)	9 (31.0)	11 (28.2)	124 (34.7)
*T. mutans*	2 (7.4)	1 (3.6)	3 (15.8)	5 (10.0)	3 (10.0)	7 (9.5)	0	1 (5.3)	5 (9.4)	2 (6.9)	1 (2.6)	30 (8.4)
*E. ruminantium*	0	5 (17.9)	8 (42.1)	8 (16.0)	3 (10.0)	6 (8.1)	0	0	0	0	3 (7.7)	33 (9.2)
**Total ticks**	**27 (6.6)**	**28 (6.9)**	**23 (6.4)**	**50 (12.3)**	**36 (10.1)**	**74 (18.1)**	**40 (9.8)**	**19 (4.7)**	**53 (13.0)**	**29 (7.0)**	**39 (9.5)**	**357 (100)**

### Co-infections of tick-borne pathogen in ticks

Multiple TBPs of up to three were detected in this study, with 3.9% (14/357) of the ticks being co-infected. Of the co-infections, 35.7% (5/14) comprised *A. marginale* and *T. mutans* in *R. microplus* ticks from Lilongwe district, while another 35.7% (5/14) comprised *A. marginale* and *T. parva* in *R. appendiculatus*, and 28.6% (4/14) comprised *T. parva* and *B. bigemina* in *R. decoloratus* from Kasungu and Lilongwe districts.

### Phylogenetic analysis of detected tick-borne pathogens

**Figure 2:**
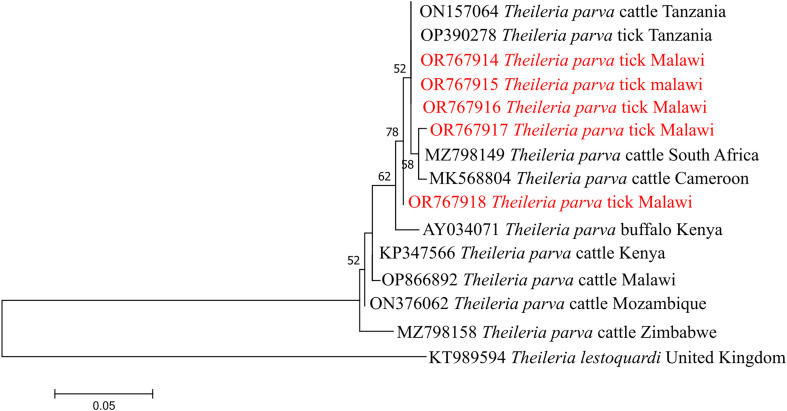
Phylogenetic analysis of *Theileria parva* based on the *p104* gene. The Maximum Likelihood method was used to construct the tree on MEGA 11 software with the Hasegawa Kishino Yano parameter model. All bootstrap values >50% from 1,000 replicates are shown on the branch nodes. Sequences from this study are indicated in red and *Theileria lestoquardi* (KT989594) was used as an outgroup.

Phylogenetic analysis of *Theileria parva* p104 gene sequences showed that all sequences in Malawi clustered within a single clade together with reference sequences from East and Southern Africa, including Tanzania, Kenya, Mozambique, and South Africa (Fig. 2).

**Figure 3:**
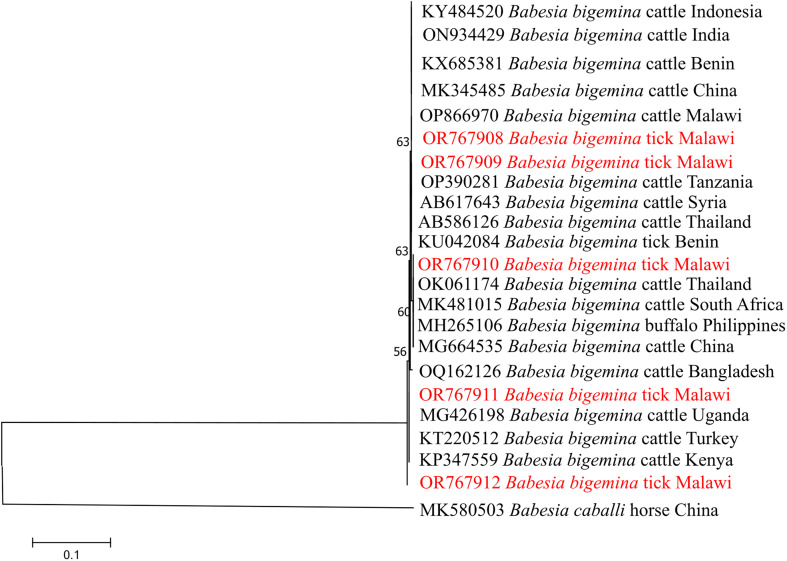
Phylogenetic analysis of *Babesia bigemina* based on the *rap-1a* gene. The Maximum Likelihood method was used to construct the tree on MEGA 11 software with the kimura 2-parameter model. All bootstrap values >50% from 1,000 replicates are shown on the branch nodes. Sequences from this study are indicated in red and *Babesia caballi* (MK580503) was used as an outgroup.

**Figure 4:**
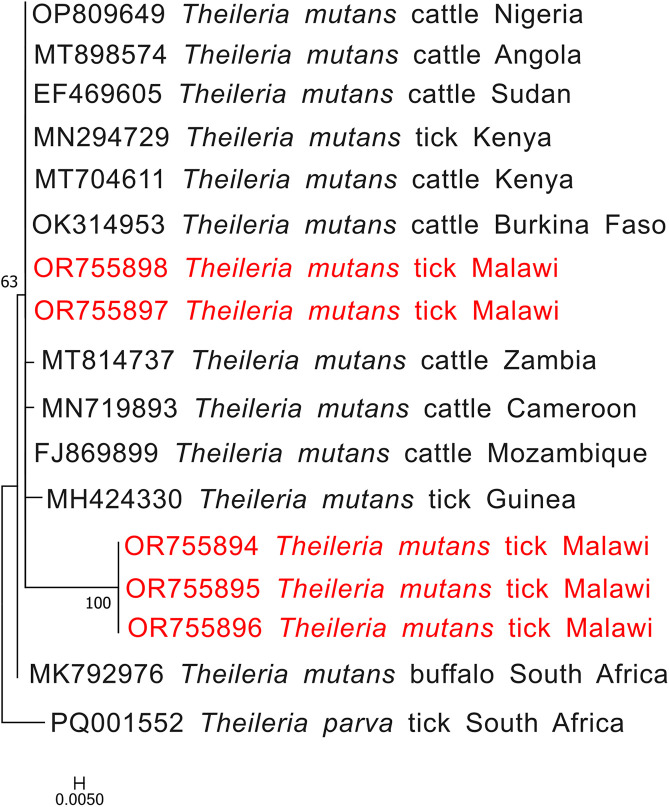
Phylogenetic analysis of *Theileria mutans* based on the 18S rRNA gene. The Maximum Likelihood method was used to construct the tree on MEGA 11 software with the TG2 parameter model. All bootstrap values >50% from 1,000 replicates are shown on the branch nodes. Sequences from this study are indicated in red and *Plasmodium falciparum* (JQ627150) was used as an outgroup.

*Babesia bigemina rap-1a* sequences grouped closely with isolates from Uganda, Kenya, South Africa, and Bangladesh, forming a conserved and well-supported clade with minimal genetic divergence (Fig. 3). Similarly, *Theileria mutans* 18S rRNA sequences clustered with reference sequences from Angola, Cameroon, Mozambique, Zambia, and South Africa, indicating widespread circulation of closely related strains across the region (Fig. 4).

**Figure 5:**
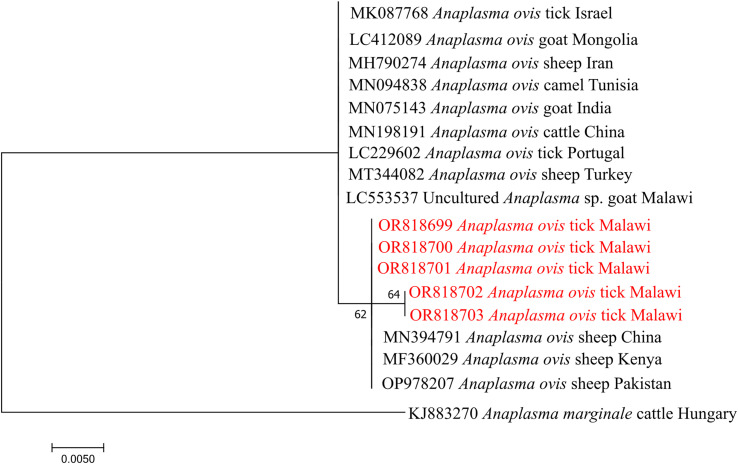
Phylogenetic analysis of *Anaplasma ovis* based on the *msp4* gene. The Maximum Likelihood method was used to construct the tree on MEGA 11 software with the Kimura-2 parameter model. All bootstrap values >50% from 1,000 replicates are shown on the branch nodes. Sequences from this study are indicated in red and *Anaplasma marginale* (KJ883270) was used as an outgroup.

**Figure 6:**
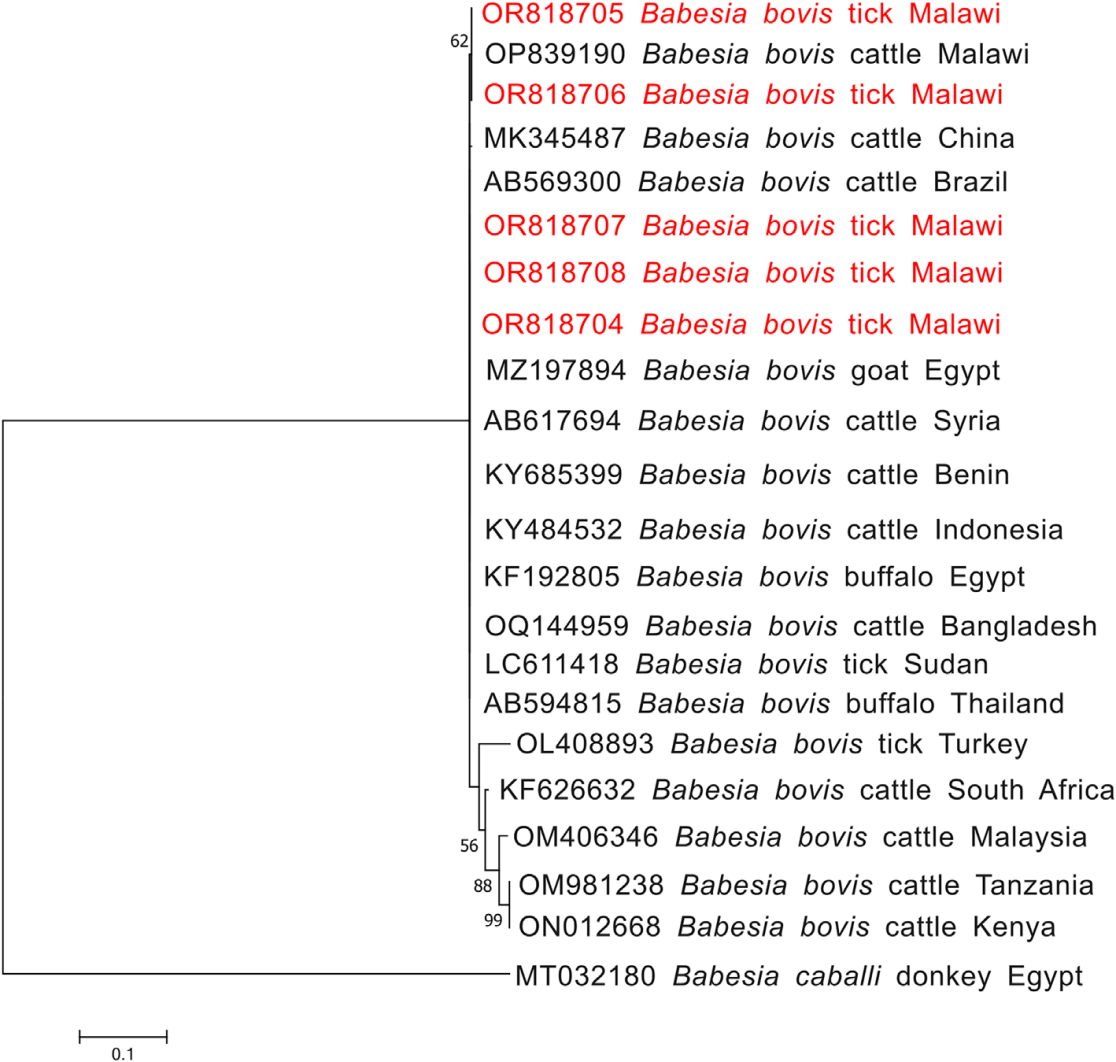
Phylogenetic analysis of *Babesia bovis* based on the *sbp4* gene. The Maximum Likelihood method was used to construct the tree on MEGA 11 software with the kimura 2-parameter model. All bootstrap values >50% from 1,000 replicates are shown on the branch nodes. Sequences from this study are indicated in red. *Babesia caballi* (MT032180) was used as an outgroup.

Phylogenetic construction of *Anaplasma ovis* based on the msp4 gene showed clustering with isolates from East Africa and Asia, including Kenya, China, and Pakistan (Fig. 5). *Babesia bovis* SBP-4 sequences formed two distinct lineages: one clustering with isolates from China and another grouping with Sudan, Egypt, Benin, Bangladesh and Syria, suggesting the presence of at least two circulating lineages in Malawi (Fig. 6).

**Figure 7:**
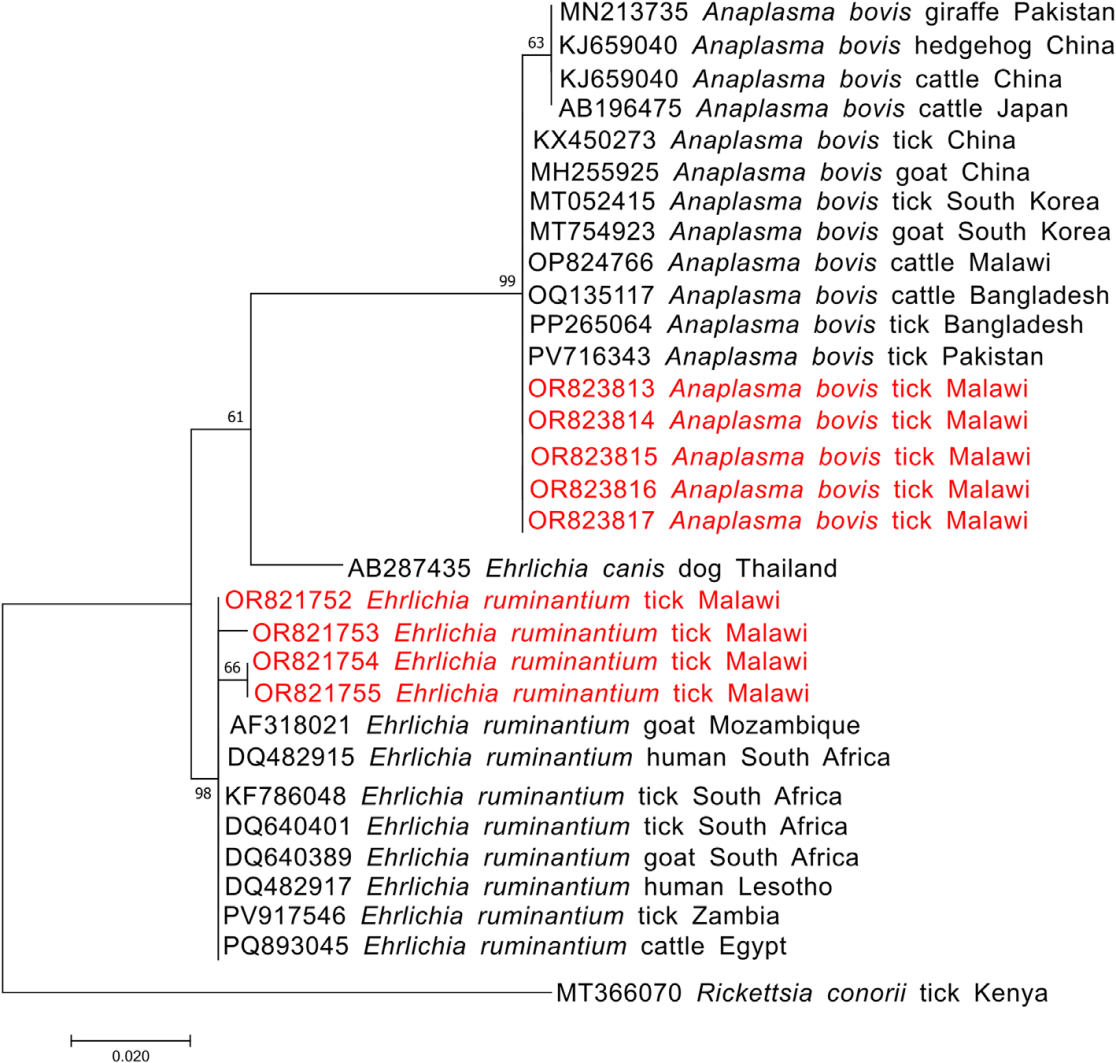
Phylogenetic analysis of *Anaplasma bovis* and *Ehrlichia ruminantium* based on the 16S rRNA gene. The Maximum Likelihood method was used to construct the tree on MEGA 11 software with the kimura 2-parameter model. All bootstrap values >50% from 1,000 replicates are shown on the branch nodes. Sequences from this study are indicated in red and *Rickettsia conorii* (MT366070) was used as an outgroup.

Analysis of 16S rRNA sequences identified as *Ehrlichia ruminantium* showed clustering with reference strains from Mozambique, South Africa, Zambia, and Egypt (Fig. 7). While sequences of *Anaplasma bovis* clustered separately within the same tree (Fig. 7).

**Figure 8:**
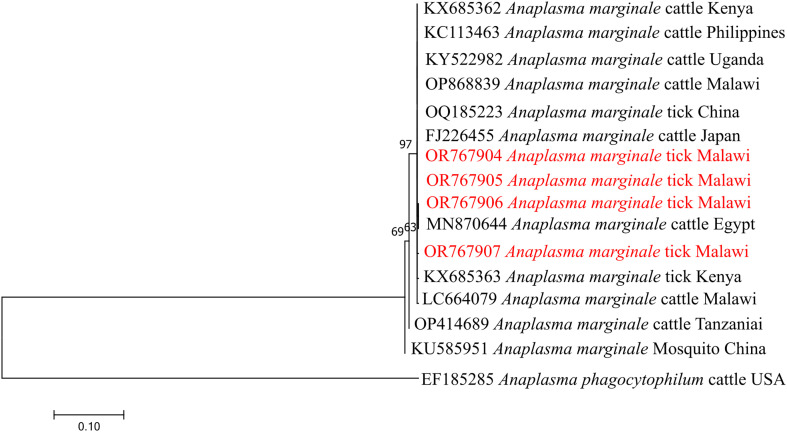
Phylogenetic analysis of *Anaplasma marginale* based on the *groEL* gene. The Maximum Likelihood method was used to construct the tree on MEGA 11 software with the kimura 2-parameter model. All bootstrap values >50% from 1,000 replicates are shown on the branch nodes. Sequences from this study are indicated in red and *Anaplasma phagocytophilum* (EF185285) was used as an outgroup.

A maximum likelihood phylogenetic tree was constructed based on partial *groEL* gene sequences to confirm the identity of *Anaplasma marginale* detected in ticks (Fig. 8). All sequences generated in this study clustered within the *A. marginale* clade together with reference sequences from Africa and other regions. The tick-derived sequences from Malawi formed a well-supported cluster (bootstrap support = 97%) closely related to reference *A. marginale* sequences from cattle in Kenya, Uganda, Egypt, and Malawi. No clear separation according to host species or geographic origin was observed within the *A. marginale* clade.

## Discussion

This study presents the first molecular-based identification and characterization of TBPs associated with ruminant-infesting ticks in Malawi. Tick species were initially identified morphologically using stereomicroscopy and taxonomical keys and confirmed using molecular tools. Accurate tick identification is fundamental in understanding and controlling TBDs, as it informs risk assessments, surveillance strategies, and vector management programs [12, 14]. Furthermore, tick species distribution mapping contributes to the understanding of ecological health and biodiversity with related conservation implications [15].

Ticks have frequently been observed on livestock in Malawi, but there is scarcity of scientific literature on the identity of these ticks and the pathogens they potentially carry. We report the identification of eleven tick species belonging to the three genera *Amblyomma*, *Hyalomma*, and *Rhipicephalus* from domestic ruminants. The identified tick species included *A. variegatum*, *R. appendiculatus*, *R. annulatus*, *R. decoloratus*, *R. evertsi*, *R. microplus*, *R. pravus*, *R. sanguineus* s. l., *R. simus*, *H. truncatum*, and *H. rufipes*. Except for *R. annulatus*, which was only found in Ntcheu and Lilongwe, all other species were detected across all sampling districts (Fig. 1), suggesting a wide distribution of tick species on domestic ruminants throughout Malawi. This finding supports earlier reports of rich tick diversity in the country [4, 7]. The uncovering of *R. appendiculatus* in the southern region is epidemiologically significant because the region was long considered vector-free, despite earlier molecular detection of *T. parva* in cattle blood [6, 8]. This finding raises an important question about how the species was introduced into this region. This may be as a result of the movement of cattle from neighboring regions where *R. appendiculatus* is well established and widely distributed, such as the central region in Malawi [4] or neighboring endemic countries such as Mozambique [4, 48]. Further, unregulated or informal livestock trade, seasonal transboundary grazing and restocking of cattle following disease outbreaks or natural disasters may have facilitated the spread of this species. Despite cattle being primary hosts of *R. appendiculatus*, incidental carriages on goats and sheep may also contribute to its local dissemination. Whether the presence of this species reflects a recent introduction or a previously undetected, low-density population cannot be conclusively determined from present data. Our findings provide the missing vector link and suggest that East Coast fever transmission risk in southern Malawi may have been underestimated.

The study revealed that *R. microplus* was the most dominant species and accounted for 30.5% of all ticks identified and was present on all host types and across all regions. This is consistent with its known capacity to infest a wide range of hosts, including wild and domestic ruminants [11, 16, 48]. In contrast, *T. parva* was the most prevalent pathogen detected across all regions of Malawi. This observation was unexpected and may indicate a recent or previously undocumented presence of *R. appendiculatus* in southern Malawi, a region historically regarded as free of this vector, with uncontrolled livestock movement representing a possible contributing factor.

The brown ear tick, *R. appendiculatus*, is typically associated with areas of tall vegetation and higher rainfall [48], and it prefers large hosts such as cattle [19, 20]. In this study, we observed the highest *R. appendiculatus* tick abundance in cattle (72.6%) followed by goats (18.2%), and sheep (9.2%). The finding of *R. appendiculatus* across all regions of Malawi contradicts earlier reports that suggested its distribution was largely confined to wetter ecological zones [4]. This broader occurrence may reflect changes in tick distribution patterns, potentially influenced by increased livestock movement across regions. As *R. appendiculatus* is the principal vector of *T. parva*, its detection in this study, in association with other tick-borne pathogens, including *A. marginale*, *B. bigemina*, *A. ovis*, and *B. bovis*, raises the possibility of co-infections in cattle. Similar patterns of multi-pathogen circulation have been documented elsewhere and have showed important implications for disease dynamics and control strategies [5, 17].

*Rhipicephalus decoloratus*, a well-established vector of *B. bigemina*, *A. marginale*, and other tick-borne pathogens [24, 36], was widely detected in this study, indicating its continued epidemiological relevance in Malawi. Its higher occurrence in cattle (81.2%) compared to goats (13.6%) and sheep (5.2%) is consistent with its known host preference and feeding behavior, and mirrors patterns reported from South Africa and Lesotho [30, 44]. The detection of multiple pathogens, including *T. parva*, *B. bigemina*, and *T. mutans*, in *R. decoloratus* further underscores its potential role in maintaining and transmitting diverse pathogens within mixed livestock systems [12]. These findings highlight the importance of considering *R. decoloratus* in integrated tick control strategies, particularly in cattle-dominated production systems where the risk of multi-pathogen exposure may be high.

The detection of *R. evertsi*, a tick species commonly associated with *T. separata* and *A. marginale* [48], further highlights the diversity of tick species infesting domestic ruminants in Malawi. Its occurrence across all study regions and predominance on cattle (100% infestation), is consistent with reports from other African settings [29, 44], reflecting its known host preference and wide ecological adaptability. The identification of *A. marginale*, *A. ovis*, *T. mutans*, and *T. parva* in *R. evertsi* suggests frequent exposure of this tick species to multiple pathogens circulating in livestock populations. However, the detection of *T. mutans* and *T. parva* in *R. evertsi* most likely reflects pathogen acquisition during blood feeding rather than confirmed vector competence, as biological transmission of these parasites by this tick species has not been conclusively demonstrated.

*Rhipicephalus annulatus* is primarily a cattle tick [48] and was only identified in goats in this study. While it is typically associated with *B. bovis* and *B. bigemina*, we detected *A. bovis* in *R. annulatus* and *R. simus* ticks. *Anaplasma bovis* has previously been detected in several *ixodid* tick species including *R*. *microplus* [25], *Haemaphysalis longicornis*, and *Ixodes persulcatus* [26]. Our finding of *A. bovis* in *R. annulatus* ticks was surprising given the limited documentation on this association. Similarly, *R. sanguineus* s. l., usually found on dogs, was detected infesting cattle and found to carry *A. marginale*. This suggests cross-species infestation possibly due to cohabitation of domestic dogs and livestock [29]. *Rhipicephalus simus* and *R. pravus* were also identified and found to carry *T. parva* and *T. mutans*, respectively, again suggesting pathogen acquisition during blood meal.

*Hyalomma rufipes*, a two-host tick and known vector of Crimean-Congo hemorrhagic fever and Nairovirus [40, 41], babesiosis [36], anaplasmosis and theileriosis [27], showed an infestation rate of 7.5%, mostly in cattle (88.5%), followed by goats (11.1%), and sheep (0.4%). The highest prevalence was attained in the southern region (73.4%), followed by the central (19.7%) and northern regions (6.9%). As observed by Zhou *et al.* [50], regional variation in prevalence suggests ecological and climatic influences. Detection of *A. marginale* further confirms its role in anaplasmosis transmission.

*Hyalomma truncatum*, the banded-legged tick, which has been implicated in the transmission of several pathogens and is associated with conditions such as dermatophilosis and Nairobi sheep disease [2, 3, 10], was detected in ticks across all ruminant species examined in this study. Although its overall occurrence was relatively low, its predominance in cattle and goats suggests host-related differences in exposure or suitability, which may reflect local husbandry practices or ecological conditions. This host distribution is inconsistent with findings from other settings where higher infestation levels were reported in sheep and lower levels in cattle [30], highlighting the influence of regional differences in livestock composition and management. The frequent detection of *A. ovis* and *E. ruminantium* in *H. truncatum* indicates substantial exposure of this tick species to circulating pathogens and suggests a potential epidemiological role, although further studies are required to confirm its vector competence under local conditions.

*Amblyomma variegatum*, the tropical bont tick, is a well-documented vector of *E. ruminantium* and has also been associated with *T. mutans* transmission [1, 2]. Its detection across all study regions, with a higher occurrence in northern Malawi (44.9%), is consistent with the ecology of this tick species, which favors warmer and more humid environments. The relatively frequent detection of *E. ruminantium* in *A. variegatum* in this study aligns with the established epidemiology of heartwater disease in sub-Saharan Africa [1, 2], reinforcing the continued importance of this tick species in the maintenance and transmission of the pathogen within ruminant populations.

Notably, this study demonstrates that several tick-borne pathogens, including *T. parva*, *A. marginale*, *B. bigemina*, and *A. ovis*, were detected across multiple tick species, highlighting the complexity of pathogen circulation within ruminant-associated tick communities. The widespread detection of *A. marginale* in six tick species supports earlier reports of its broad vector range and ecological adaptability [36]. Similarly, the occurrence of *B. bigemina* in both *R. appendiculatus* and *R. decoloratus* indicates exposure of multiple tick species to this pathogen and suggests potential overlap in transmission cycles, which may increase the risk of co-infections in cattle populations [32].

The phylogenetic patterns observed in this study indicate that several TBPs circulating in Malawi are genetically closely related to strains reported across diverse regions of Africa and beyond, suggesting broad regional connectivity and limited geographic structuring for some loci. For *T. parva*, the *p104* gene sequences clustered within a single major clade together with isolates from Tanzania (OP390278 and ON157064), South Africa (MZ798149), Cameroon (MK568804), Kenya (AY034071), Mozambique (ON376062), and Malawi (OP866892), consistent with previous reports [37], showing limited polymorphism in this marker and an absence of strong geographic segregation (Fig. 2). This finding aligns with earlier studies [37], indicating that the *p104* gene is relatively conserved and therefore useful for confirming *T. parva* infection, but has limitation in resolving strain-level variation. This phenomenon supports the application of the *p104* gene in East Coast fever routine surveillance, while emphasizing the need for complementary markers to inform epidemiological studies.

Similarly, *B. bigemina* sequences based on the *rap-1a* gene clustered with isolates from East Africa (Uganda, MG426198, and Kenya, KP347559), Southern Africa (South Africa, MK481015), and Asia (Bangladesh, OQ162126), indicating the wide geographic distribution and genetic homogeneity (Fig. 3). The clustering of *T. mutans* 18S rRNA sequences with those from multiple African countries including Angola, Cameroon, Mozambique, South Africa, Burkina Faso, and Zambia (Fig. 4), further supports the notion of widespread circulation of closely related strains across the continent, likely facilitated by livestock movement and shared tick vectors.

In contrast, the phylogenetic structure of *B. bovis* suggested greater genetic complexity. While most Malawian sequences clustered with isolates from Sudan (LC611418), Bangladesh (OQ144959), and Brazil (AB569300), one sequence formed a distinct lineage with a previously Malawian isolate (OP839190), indicating the possible presence of at least two circulating *B. bovis* lineages in Malawi (Fig. 6). This finding has important epidemiological implications, as genetic heterogeneity in *B. bovis* has been associated with variation in virulence and diagnostic sensitivity [38].

The clustering of *A. ovis* major surface protein 4 (msp4) sequences with isolates from Asia (Pakistan, OP978207 and China, MN394791), and East Africa (Kenya, MF360029) (Fig. 5), suggesting that there might be one strain circulating in Malawi. The 16S rRNA *Ehrlichia ruminantium* sequences from this study clustered within the *Ehrlichia ruminantium* clade together with reference strains from other African countries. Therefore, based on the phylogenetic placement and sequence similarity, these sequences are interpreted as *E. ruminantium* sensu stricto and not closely related to *Ehrlichia* taxa (Fig. 7).

The *groEL*-based phylogenetic analysis further supports the molecular identification of *Anaplasma marginale* detected in ticks collected from livestock in Malawi. The clustering of sequences from Malawi with reference *A. marginale* sequences from multiple regions, including East and North Africa, indicates that the detected genotypes are consistent with globally distributed *A. marginale* lineages. The absence of strong geographic or host-associated structuring within the *A. marginale* clade is consistent with previous studies [13, 18, 27], and likely reflects the broad host range and widespread distribution of this pathogen. Although the *groEL* gene provides useful resolution for species confirmation, additional markers [17] and broader sampling would be required to assess fine-scale genetic diversity and epidemiologic patterns of *A. marginale* in the region.

Collectively, these findings suggest that TBPs in Malawi form part of wider regional and global transmission networks, underscoring the need for coordinated surveillance strategies and the use of higher-resolution markers to better resolve population structure and transmission dynamics.

The detection of a pathogen in a tick species does not necessarily demonstrate vector competence as biological transmission requires successful pathogen replication and passage to a susceptible host [15], with molecular evidence to be interpreted and vector competence established through rigorous experimental infection and transmission studies.

In conclusion, this study provides the first molecular and morphological evidence of TBPs in ticks infesting domestic ruminants in Malawi. The findings highlight the presence of multiple pathogens across different tick species, underscoring the importance of integrated tick and TBD control strategies. The finding and identification of *R. appendiculatus* in southern Malawi, a region where it was previously considered absent, together with the detection of *T. parva*, raises important concerns for East Coast fever surveillance and control. These observations indicate the need for a re-evaluation of current monitoring and control programs, particularly considering potential changes in tick distribution and the emergence of new vector-pathogen associations.

## Supplementary material

*Table S1*. Number of ticks collected by developmental stage, tick species, and host animal infesting domestic ruminants in Malawi. The supplementary material of this article is available at https://www.parasite-journal.org/10.1051/parasite/2026022/olm.

## References

[1] Allsopp BA. 2010. Natural history of *Ehrlichia ruminantium*. Veterinary Parasitology, 167(2), 123–135. 19836892 10.1016/j.vetpar.2009.09.014

[2] Allsopp BA. 2015. Heartwater-*Ehrlichia ruminantium* infection. Revue Scientifique et Technique de l’OIE, 34(2), 557–568. 10.20506/rst.34.2.2379 26601456

[3] Ambrose N, Lloyd D, Maillard JC. 1999. Immune responses to *Dermatophilus congolensis* infections. *Parasitology Today*, 15 (7), 295–300. 10377534 10.1016/s0169-4758(99)01470-2

[4] Berggren SA. 1978. Cattle ticks in Malawi. Veterinary Parasitology, 4 (3), 289–297.

[5] Bockenstedt LK, Gonzalez DG, Haberman AM, Belperron AA. 2012. Spirochete antigens persist near cartilage after murine Lyme borreliosis therapy. Journal of Clinical Investigation, 122 (7), 2652–2660. 22728937 10.1172/JCI58813 PMC3386809

[6] Chatanga E, Maganga E, Mohamed WMA, Ogata S, Pandey GS, Abdelbaset AE, Hayashida K, Sugimoto C, Katakura K, Nonaka N, Nakao R. 2022. High infection rate of tick-borne protozoan and rickettsial pathogens of cattle in Malawi and the development of a multiplex PCR for *Babesia* and *Theileria* species identification. Acta Tropica, 231, 106413. 35307457 10.1016/j.actatropica.2022.106413

[7] Chikufenji B, Chatanga E, Galon EM, Mohanta UK, Mdzukulu G, Nkhata M, Ma Y, Shirafuji-Umemiya R, Xuan X. 2024. First report of dog ticks and tick-borne pathogens they are carrying in Malawi. Journal of Veterinary Medical Science, 86 (2), 150–159. 38171881 10.1292/jvms.23-0397 PMC10898992

[8] Chikufenji B, Galon EM, Chatanga E, Kamanga N, Mohanta UK, Ma Z, Hayashida K, Xuan X. 2024. Molecular detection and phylogenetic analysis of tick-borne pathogens in cattle in southern Malawi. Veterinary Research Communications. 48, 2753–2760. 38676858 10.1007/s11259-024-10395-z

[9] Chinombo DO, Mzoma FJ, Musisi FL. 1988. East Coast fever in Malawi (1983–1987).Theileriosis in Eastern Central and Southern Africa, in Proceedings of a Workshop on ECF Immunization Lilongwe Malawi, Dolan TT, Editor. ILRAD: Nairobi Kenya. pp. 12–16.

[10] Davies FG. 1978. Nairobi sheep disease in Kenya. The isolation of viruses from sheep and goats, ticks and possible maintenance hosts. Journal of Hygiene, 81(2), 257–265. 10.1017/s0022172400025092 PMC2129769 701790

[11] De Castro JJ. 1997. Sustainable tick and tick-borne disease control in livestock improvement in developing countries. Veterinary Parasitology, 71 (2), 77–97. 9261972 10.1016/s0304-4017(97)00033-2

[12] De la Fuente J, Estrada-pena A, Venzal JM, Kocan KM, Sonenshine DE. 2008. Overview: Ticks as vectors of pathogens that cause disease in humans and animals. Frontiers of Bioscience, 13 (18), 6938–6946. 10.2741/3200 18508706

[13] De la Fuente J, Torina A, Naranjo V, Caracappa S, Vicente J, Mangold AJ, Vicari D, Alongi A, Scimeca S, Kocan KM. (2005). Genetic diversity of *Anaplasma marginale* strains from different geographic locations. Journal of Veterinary Medicine, 52 (5), 226–229. 16115096 10.1111/j.1439-0450.2005.00851.x

[14] Eisen RJ, Eisen L. 2018. The blacklegged tick, *Ixodes scapularis*: an increasing public health concern. Trends in Parasitology, 34 (4), 295–309. 29336985 10.1016/j.pt.2017.12.006 PMC5879012

[15] Estrada-Pena A, de la Fuente J. 2014. The ecology of ticks and epidemiology of tick-borne, viral diseases. Antiviral Research, 108, 104–128. 24925264 10.1016/j.antiviral.2014.05.016

[16] George JE, Pound JM, Davey RB. 2004. Chemical control of ticks on cattle and the resistance of these parasites to acaricides. Parasitology, 129 (1), 353–366. 10.1017/s0031182003004682 15938518

[17] Guo WP, Wang X, Li YN, Xu G, Wang YH, Zhou EM. (2019). GroEL gene typing and genetic diversity of *Anaplasma bovis* in ticks in Shaanxi, China. Infection, Genetics and Evolution, 74, 103927. 10.1016/j.meegid.2019.103927 31220612

[18] Hailemariam Z, Krücken J, Baumann M, Ahmed JS, Clausen PH, Nijhof AM. 2017. Molecular detection of tick-borne pathogens in cattle from Southwestern Ethiopia. PLoS One, 12 (11), e0188248. 29155863 10.1371/journal.pone.0188248 PMC5695778

[19] Horak IG, Heyne H, Halajian A, Booysen S, Smit WJ. 2017. Parasites of domestic and wild animals in South Africa. L. *Ixodid* ticks infesting horses and donkeys. Onderstepoort Journal of Veterinary Research, 84 (1), a1301. 10.4102/ojvr.v84i1.1302 PMC6238695 28281774

[20] Horak IG, Nyangiwe N, De Matos C, Neves L. 2009. Species composition and geographic distribution of ticks infesting cattle, goats and dogs in a temperate and in a subtropical region of south-East Africa. Onderstepoort Journal of Veterinary Research, 76 (3), 263–276. 21105593 10.4102/ojvr.v76i3.28

[21] Inokuma H, Oyamada M, Kelly PJ, Jacobson LA, Fournier PE, Itamoto K, Okuda M, Brouqui P. 2005. Molecular detection of a new *Anaplasma* species closely related to *Anaplasma phagocytophilum* in canine blood from South Africa. Journal of Clinical Microbiology, 43 (6), 2934–2937. 15956424 10.1128/JCM.43.6.2934-2937.2005 PMC1151900

[22] Jongejan F, Uilenberg G. 2004. The global importance of ticks. Parasitology, 129(1), S3–S14. 15938502 10.1017/s0031182004005967

[23] Jonsson NN. 2006. The productivity effects of cattle tick (*Boophilus microplus*) infestation on cattle, with particular reference to *Bos indicus* cattle and their crosses. Veterinary Parasitology, 137 (1), 1–10. 16472920 10.1016/j.vetpar.2006.01.010

[24] Kocan KM, de la Fuente J, Blouin EF, Coetzee JF. 2010. The natural history of *Anaplasma marginale*. *Veterinary Parasitology*, 167 (2), 95–107. 19811876 10.1016/j.vetpar.2009.09.012

[25] Kawahara M., Rikihisa Y., Lin Q., Isogai E., Tahara K., Itagaki A., Hiramitsu Y., Tajima T. 2006. Novel genetic variants of *Anaplasma phagocytophilum*, *Anaplasma bovis*, *Anaplasma centrale*, and a novel *Ehrlichia* sp. in wild deer and ticks on two major islands in Japan. Applied and Environmental Microbiology. 72 (2), 1102–1109. 16461655 10.1128/AEM.72.2.1102-1109.2006 PMC1392898

[26] Kim CM, Kim MS, Park MS, Park JH, Chae JS. 2003. Identification of *Ehrlichia chaffeensis*, *Anaplasma phagocytophilum*, and *A. bovis* in *Haemaphysalis longicornis* and *Ixodes persulcatus* ticks from Korea. Vector-Borne and Zoonotic Diseases. 3 (1), 17–26 12804377 10.1089/153036603765627424

[27] Kocan KM, de la Fuente J, Guglielmone AA. 2003. Antigens and alternatives for control of *Anaplasma marginale* infection in cattle. Clinical Microbiology Reviews, 16 (4), 698–712. 14557295 10.1128/CMR.16.4.698-712.2003 PMC207124

[28] Latrofa MS, Dantas-Torres F, Giannelli A, Otranto D. 2014. Molecular detection of tick-borne pathogens in *Rhipicephalus sanguineus* group ticks. Ticks and Tick-Borne Diseases, 5 (6), 943–946. 25113982 10.1016/j.ttbdis.2014.07.014

[29] Madder M, Horak I, Stoltsz H. 2010. Ticks: tick identification. University of Pretoria, Faculty of Veterinary Science, Pretoria, South Africa.

[30] Mahlobo-Shwabede SIC, Zishiri OT, Thekisoe OMM, Bakkes D, Bohloa L, Molomo M, Makalo MJR, Mahloane GR, Mtshali MS. 2022. Ticks of domestic animals in Lesotho: Morphological and molecular characterization. Veterinary Parasitology Regional Studies and Reports, 29, 100691. 35256119 10.1016/j.vprsr.2022.100691

[31] Meltzer MI, Norval RAI, Donachie PL. 1995. Effects of tick infestation and tick-borne disease infections (heartwater, anaplasmosis and babesiosis) on the lactation and weight gain of mashona cattle in south-eastern Zimbabwe. Tropical Animal Health and Production, 27, 129–144. 7502343 10.1007/BF02248956

[32] Minjauw B, McLeod A. 2003. Tick-borne diseases and poverty. The impact of ticks and tick-borne diseases on the livelihoods of small-scale and marginal livestock owners in India and eastern and southern Africa. Research Report, DFID Animal Health Programme, Centre for Tropical Veterinary Medicine, University of Edinburgh.

[33] Ministry of Agriculture, Malawi. 2023. Third round agricultural production estimates (APES) 2022/2023 fiscal year. Department of Animal Health and Livestock Development, Lilongwe, Malawi.

[34] Moyo B, Masika PJ. 2009. Tick control methods used by resource-limited farmers and the effect of ticks on cattle in rural areas of the Eastern Cape Province, South Africa. Tropical Animal Health and Production, 41, 517–523. 18704741 10.1007/s11250-008-9216-4

[35] Mtshali K, Khumalo ZTH, Nakao R, Grab DJ, Sugimoto C, Thekisoe OMM. 2016. Molecular detection of zoonotic tick-borne pathogens from ticks collected from ruminants in four South African provinces. Journal of Veterinary Medical Science, 77 (12), 1573–1579. 26227797 10.1292/jvms.15-0170 PMC4710712

[36] Mtshali K, Mtshali PS. 2013. Molecular diagnosis and phylogenetic analysis of *Babesia bigemina* and *Babesia bovis* hemoparasites from cattle in South Africa. BMC Veterinary Research, 9, 154. 23927555 10.1186/1746-6148-9-154 PMC3751629

[37] Obara I, Makori P, Sibeko KP. et al. 2023. Conservation and variation in the region of the *Theileria parva* p104 antigen coding gene used for PCR surveillance of the parasite. Parasitology Research*,* 122, 1381–1390. 37081209 10.1007/s00436-023-07838-y PMC10172223

[38] Odongo DO, Sunter JD, Kiara HK, Skilton RA, Bishop RP. 2010. A nested PCR assay exhibits enhanced sensitivity for detection of *Theileria parva* infections in bovine blood samples from carrier animals. Parasitology Research, 106, 357–365. 19902251 10.1007/s00436-009-1670-z

[39] Papadopoulos B, Papanastassopoulou M. 1998. Skin reactions to the bites of *Ixodid* ticks in sheep and cattle. Experimental and Applied Acarology, 22, 507–515.

[40] Parola P, Raoult D. 2001. Ticks and tickborne bacterial diseases in humans: an emerging infectious threat. Clinical Infectious Diseases, 32 (6), 897–928. 11247714 10.1086/319347

[41] Peter RJ, Van den Bossche P, Penzhorn BL, Sharp B. 2005. Tick, fly, and mosquito control-lessons from the past, solutions for the future. Veterinary Parasitology, 132 (3), 205–215. 16099104 10.1016/j.vetpar.2005.07.004

[42] Simuunza M, Weir W, Courcier E, Tait A, Shiels B. 2011. Epidemiological analysis of tick-borne diseases in Zambia. Veterinary Parasitology, 175 (3), 331–342. 21106294 10.1016/j.vetpar.2010.09.027

[43] Snellgrove AN, Krapiunaya I, Ford SL, Stanley HM, Wickson AG, Hartzer KL, Levin ML. 2020. Vector competence of *Rhipicephalus sanguineus* sensu stricto for *Anaplasma platys*. Ticks and Tick-Borne Diseases. 11 (6), 101517. 32993937 10.1016/j.ttbdis.2020.101517 PMC7571858

[44] Spickett AM, Heyne IH, Williams R. 2011. Survey of the livestock ticks of the north-west province, South Africa. Onderstepoort Journal of Veterinary Research 78 (1), a305. 10.4102/ojvr.v78i1.305 23327212

[45] Swanepoel R, Burt FJ. 2004. Crimean–Congo hemorrhagic fever. Second Edition, in Infectious diseases of livestock with special reference to South Africa, Coetzer JAW, Tustin RC, Editors. Cape Town: Oxford University Press Southern Africa. pp. 1077–1085.

[46] Tamura K, Stecher G, Kumar S. 2021. MEGA11: Molecular Evolutionary Genetics Analysis Version 11. Molecular Biology and Evolution, 38 (7), 3022–3027. 33892491 10.1093/molbev/msab120 PMC8233496

[47] Terkawi MA, Huyen NX, Shinuo C, Inpankaew T, Maklon K, Aboulaila M, Ueno A, Goo YK, Yokoyama N, Jittapalapong S, Xuan X, Igarashi I. 2011. Molecular and serological prevalence of *Babesia bovis* and *Babesia bigemina* in water buffaloes in the northeast region of Thailand. Veterinary Parasitology, 178(3), 201–207. 21324601 10.1016/j.vetpar.2011.01.041

[48] Walker A, Estrada-Pena A, Bouattour A, Horak I. 2003. Ticks of Domestic Animals in Africa: a guide to Identification of Species. Edinburgh, UK: Bioscience Reports.

[49] Ybañez AP, Ybañez RH, Claveria FG, Cruz-Flores MJ, Xuan X, Yokoyama N, Inokuma H. 2014. High genetic diversity of *Anaplasma marginale* detected from Philippine cattle. Journal of Veterinary Medical Science, 76 (7), 1009–1014. 24717413 10.1292/jvms.13-0405 PMC4143641

[50] Zhou R, Sprong H, Liu Q, Krafft T, Estrada-Peña A. 2025. Mapping the potential suitable habitats for *Hyalomma rufipes* (Acari: Ixodidae) in Africa and Western Eurasia. PLoS Neglected Tropical Diseases, 19 (3), e0012923. 40100878 10.1371/journal.pntd.0012923 PMC11918435

